# Gastrointestinal (GI)-Specific patient reported outcomes instruments differentiate between renal transplant patients with or without GI symptoms: results from a South American cohort

**DOI:** 10.1186/1477-7525-6-53

**Published:** 2008-07-21

**Authors:** Gerardo Machnicki, Jacqueline Pefaur, Luis Gaite, Ana M Linchenco, Clemente Raimondi, Ruben Schiavelli, Alcira Otero, Mary Kay Margolis

**Affiliations:** 1 Global Health Economics and Outcomes Research, Novartis Pharmaceuticals Corp, East Hanover, NJ, USA; 2 Nephrology and Transplant, Hospital Barros Luco, Santiago, Chile; 3 Nephrology and Transplant, Clinica de Urologia y Nefrologia, Santa Fe, Argentina; 4 Nephrology and Transplant, Hospital Italiano, Rosario, Argentina; 5 Neprhology and Transplant, Hospital Español, La Plata, Argentina; 6 Nephrology and Transplant, Hospital Argerich, Buenos Aires, Argentina; 7 Medical Department, Novartis Argentina, Buenos Aires, Argentina; 8 Center for Health Outcomes Research, United BioSource Corporation, Bethesda, MD, USA

## Abstract

**Background:**

Immunosuppressive therapies have burdensome side effects which may lead to sub-therapeutic dosing and non-compliance. Patients on different immunosuppressant regimens may feel less bothered by Gastrointestinal (GI) side effects or report better health-related quality of life (HRQL). We evaluated the reliability and validity of two GI-specific outcome instruments (Gastrointestinal Symptom Rating Scale (GSRS; higher scores = increased severity) and Gastrointestinal Quality of Life Index (GIQLI; higher scores = better GI-specific HRQL)) in renal transplant patients in South America.

**Methods:**

Data from 5 South American centers participating in an international, longitudinal, observational study were analyzed. Patients were ≥ 1 month post transplant and on mycophenolate mofetil (MMF) and a calcineurin inhibitor. Patients completed the GSRS, GIQLI, and Psychological General Well-Being (PGWB; higher scores = better HRQL) Index at baseline and at 4–6 weeks. Internal consistency, test-retest reliability and construct and discriminant validity were assessed.

**Results:**

Sixty-two participants were enrolled. Mean age was 42 years; mean time since transplant was 3.3 years; 57% were male; 65% received a deceased organ transplant and 68%had GI events. The GSRS and GIQLI demonstrated high internal consistency (Cronbach's alphas 0.72–0.96). Test-retest reliability was adequate (intraclass correlation coefficient > 0.6) for all GIQLI subscales and all GSRS subscales except Diarrhea and Reflux syndrome. Correlations between the GSRS and PGWB were moderate (range: -0.21 to -0.53, all p < 0.001 except 6 correlations with p < 0.05); correlations between the GIQLI and PGWB were higher (range: 0.36 to 0.71 p < 0.001), indicating good construct validity. The GSRS and GIQLI demonstrated good discriminant validity, as they clinically and statistically distinguished between patients with and without GI complaints and among patients with varying GI complication severity. Patients with GI complaints reported higher GSRS scores than patients without complaints (all p < 0.001). GIQLI scores were lower in patients with GI complaints than patients without complaints (all p < 0.001). The GSRS and GIQLI differentiated among patients with four GI severity levels (overall Kruskall-Wallis test p < 0.001, except for one scale). The GSRS and GIQLI are acceptable for use in South American renal transplant patients. These two instruments demonstrate adequate reliability and validity. Patients with GI complaints reported poor HRQL and strategies are needed to improve patients' HRQL.

## Background

Graft survival in renal transplant patients has improved steadily over the last decades as a result of improved immunosuppressive therapies. Immunosupressive regimens continue to have side effects which patients find burdensome and which may lead to sub-therapeutic dosing and non-compliance by the patient.

Specifically, upper and lower gastrointestinal (GI) side effects such as reflux, diarrhea, and constipation are frequent occurrences with these medications. While the effects are understood on a clinical level, little is known about the patient perspective. Some evidence suggests that patients on different immunosuppressant regimens may feel less bothered by GI side effects or report better health-related quality of life (HRQL)[1]. Therefore patient reports of these GI complications and HRQL are important. Like any clinical measure, patient-reported outcomes must be valid and reliable.

The Gastrointestinal Symptom Rating Scale (GSRS) and the Gastrointestinal Quality of Life Index (GIQLI) are two patient reported outcomes instruments with demonstrated reliability and validity for use among renal transplant populations[2,3]. However, the study population did not include South American participants. Additionally test-retest reliability was not able to be assessed in the original study. The objective of this study was to evaluate the psychometric characteristics (reliability and validity) of the GSRS and GIQLI in South American patients who have had a renal transplant.

## Methods

### Study population

Data from the PROGIS study, *Measurement of Patient Reported Outcomes in Renal Transplant Patients with and without Gastrointestinal Symptoms (PROGIS)*[1] were used for this validation study. PROGIS was a longitudinal, observational study of patients post renal transplant designed to assess the impact of GI symptoms on symptom severity and HRQL and changes in these patient-reported outcomes (PROs) that occur as a result of conversion from mycophenolate mofetil (MMF) to an enteric-coated formulation of mycophenolate sodium (EC-MPS) (*myfortic*^®^). PROGIS was conducted in twenty-seven clinical sites in six countries, with a total per-protocol population of 278 patients: 177 post-transplant patients who were experiencing GI complaints and were eligible to convert to EC-MPS and 101 post-transplant patients who were not experiencing GI complaints remained on MMF. Participants were evaluated at Baseline again at 4–6 weeks. Additional details and results of the PROGIS study can be found in Chan[1]. This validation study utilized data from five sites in Argentina and Chile only.

Research was conducted according to the ICH Harmonized Tripartite Guidelines for Good Clinical Practice and in compliance with the ethical principles of the Helsinki Declaration. Appropriate Institutional Review Board/Ethics Committee approval was obtained at each center prior to study initiation. Patients were eligible for participation if they were at least 18 years of age; were willing to provide informed consent and adhere to study requirements; had received a renal transplant at least 1 month prior to the study enrollment; and had been on an immunosuppressive regimen including MMF for at least two weeks prior to enrollment. Patients were either eligible to convert to EC-MPS because of GI complaints or were not experiencing GI complaints and were stable on their current regimen. Patients were not eligible for participation if their current GI symptoms were not assumed or known to be caused by MMF; they had an episode of acute rejection less than 1 week prior to study enrollment; they were undergoing an acute medical intervention or hospitalization; they were a woman of child-bearing age not willing to use an effective means of birth control; they had a major psychiatric illness or other medical condition that might interfere with ability to complete the study; or they had received any investigational drug within 30 days prior to study enrollment.

### Assessments

Patients who met study entry criteria and provided informed consent were either converted to EC-MPS at Visit 1 (Group 1) or remained on MMF (Group 2). Participants continued on the appropriate immunosuppressant regimen (i.e., EC-MPS or MMF plus other transplant medications) as dictated by good clinical practice. Site study staff provided basic demographic and clinical information. Participants completed three self-administered questionnaires in a private room at the clinic: the Gastrointestinal Symptom Rating Scale (GSRS), the Gastrointestinal Quality of Life Index (GIQLI) and the Psychological General Well-being Index (PGWB) at Baseline. At Visit 2 the participants completed the same questionnaires, with the addition of the Overall Treatment Effect (OTE) scale.

The GSRS [4-6] is a 15-item instrument designed to assess the symptoms associated with common GI disorders. It has 5 subscales (Reflux, Diarrhea, Constipation, Abdominal Pain, and Indigestion Syndrome). Subscale scores range from 1 to 7 and higher scores represent more discomfort. The Spanish for Argentina version of the questionnaire was utilized for this study. The minimal important difference (MID) is the smallest difference in the scores that is perceived as significant by the clinician or the patient[7]. The MID range in renal transplant recipients has been calculated as a range between 0.4 for diarrhea and 0.8 for reflux[1].

The GIQLI [8] is a 36-item GI-specific HRQL instrument designed to assess HRQL in clinical practice and clinical trials of patients with GI disorders. The GIQLI has five subscales (GI Symptoms, Emotion, Physical Function, Social Function, and Medical Treatment) as well as a Total Score. Higher scores represent better HRQL and subscores range from 0–4 while the total score range from 0–144. Recent MID calculations were 12.7 for the total score, while for the subscales the range was 0.5 for Social Function to 0.2 for Emotional Status[1]. The Spanish version of the questionnaire, with minor wording modifications introduced by the research team based on well known differences in medical terms between Spain and South America, was utilized for this study.

The PGWB [9] is a 22-item measure designed to measure generic HRQL through assessment of psychological well-being and distress. The PGWB has 6 subscales (Anxiety, Depression, Positive Well-being, Self-control, General Health, and Vitality) as well as a total score. Higher scores represent better HRQL and the range is 0–100 for both the subscales and the total score. The Spanish version of the questionnaire was utilized for this study.

Additional information collected included demographic and socioeconomic data, time since transplant, type of transplant (deceased vs. living donor), current GI complications, severity of participant's GI complications (per clinician impression), concomitant immunosuppressant medication, concomitant medications which could lead to GI complaints, concomitant medications to treat or prevent GI symptoms and complications, and adverse events/infections for each participant throughout the study duration[1].

### Statistical Analyses

All analyses were performed with SAS version 8.02 (SAS Institute, Cary NC). Demographic variables and clinical conditions were evaluated by descriptive analyses. For the descriptive analyses, chi-square tests were used to evaluate categorical data; t-tests and analyses of variance (ANOVA) were used to evaluate continuous data. Scoring – including imputations for missing data if necessary – was performed according to each questionnaire's guidelines.

Reliability refers to the consistency of items within an instrument, either over time or internally within the instrument. Internal consistency reliability is the extent to which all items measure the same construct; values are presented descriptively, on an internal level scale from 0 to 1.0, with higher scores indicating a more reliable (precise) instrument. A Cronbach's alpha of 0.70 or greater indicates acceptable internal consistency reliability for an instrument used with group data[10]. The internal consistency reliability of the GSRS and GIQLI total and subscale scores was estimated using coefficient alpha.

Test-retest reliability, or reproducibility of the measure, refers to the degree to which scores remain the same over time when no change is expected [11,12]. Reproducibility assesses whether stable participants (based on responses on the OTE) scored similarly on the GSRS and GIQLI from Baseline to Visit 2. Hays and colleagues[11] suggest that intraclass correlation coefficients (ICCs) should be greater than 0.60 in stable participants.

Validity refers to the extent to which the instrument measures the construct it purports to measure and also the extent to which the instrument is useful for its intended purpose[10,11,13]. Kendall's tau correlation coefficients were used to assess construct validity. Evaluation of validity can use instruments that measure similar or dissimilar constructs. For example a disease specific HRQL instrument is often validated by using a generic instrument. However one can also evaluate validity by using instruments that measure different yet related constructs, in this case making an assumption that correlations would be lower. For this study, we used generic HRQL instruments to assess validity of both the disease-specific GIQLI as well as the GSRS, a measure of symptom impact. Construct validity focused on the pattern and magnitude of the relationship among the GSRS and GIQLI scale scores and the PGWB. We expected to find the following: 1) the relationship between the two instruments measuring HRQL (the GIQLI and PGWB) will be stronger, producing correlations of greater magnitude than that between the GSRS and PGWB; 2) the relationship between the PGWB total score and the GSRS subscales will be low to moderate (0.10 < r < 0.40); 3) the relationship between the PGWB subscales and the GSRS subscales will be low to moderate (0.10 < r < 0.50) with higher correlations being found between the GSRS Abdominal Pain and Indigestion Syndrome and all PGWB total and subscale scores compared to the remaining three GSRS subscales (Diarrhea, Constipation, Reflux Syndrome) 4) the relationship between the GIQLI total score and the PGWB total score will be moderate and significant and 5) the relationship between the GIQLI Symptom subscale and the PGWB subscales will be low to moderate (0.10 < r < 0.50) and significant[14].

Discriminant or known groups validity is the extent to which scores from an instrument are distinguishable from groups of subjects that differ by a key indicator, often clinical in nature[15]. To evaluate known groups validity, GSRS and GIQLI scores were analyzed by the presence or absence of GI complications using Wilcoxon rank-sum tests and by clinical severity (none, mild, moderate, severe) of GI complications (as rated by the clinician) using a Kruskall-Wallis test of overall differences and Wilcoxon rank-sum tests for pairwise comparisons. The expectation would be that scores on the GSRS and GIQLI would be worse for patients with GI complaints as compared to patients without GI complaints. Additionally, we expected to see worse scores for patients with more severe GI effects.

## Results

### Study Sample

Sixty-two participants were enrolled: 44 participants at four sites in Argentina and 18 participants at one site in Chile. Table 1 presents the demographic and clinical characteristics of the participants at Baseline. Participants were, on average, 42 years old, and had their transplant 3.3 years prior to study enrollment. Fifty seven percent of the participants were male. Almost two-thirds of the participants (65%) had received a deceased transplant and had GI complaints ranging from mild to severe (68%). Abdominal pain was the most frequently-reported complaint (61%); 52% reported dyspepsia, 40% diarrhea, and 34% reported nausea. None of the differences between participants were statistically significant, however, a higher proportion of patients in the test-retest sample (group 2) were male, older, with lower educational status and had shorter time since transplant. Participants were very similar to the participants in the entire PROGIS study (data not shown).

**Table 1:** Baseline Demographic and Clinical Characteristics

Characteristic	With GI events (N = 42)	Without GI events (N = 20)	Total (N = 62)	p-value for overall group difference
Age (mean, SD)	(41.8, 10.6)	(43.4, 13.0)	(42.3, 11.4)	0.617
				
Gender (n, % Male)	(22, 52.4%)	(13, 65.0%)	(35, 56.5%)	0.349
				
Domestic status (n, % yes)				0.418
Living alone	(2, 4.8%)	(2, 10.0%)	(4, 6.5%)	
Living with a partner or spouse	(35, 83.3%)	(15, 75.0%)	(50, 80.6%)	
Other	(5, 11.9%)	(2, 10.0%)	(7, 11.3%)	
Missing	(0, 0.0%)	(1, 5.0%)	(1, 1.6%)	
				
Education completed (n, % yes)				0.183
Elementary/primary school	(11, 26.2%)	(11, 55.0%)	(22, 35.5%)	
Secondary/high school	(18, 42.9%)	(4, 20.0%)	(22, 35.5%)	
College degree	(8, 19.0%)	(4, 20.0%)	(12, 19.4%)	
Post-graduate degree	(3, 7.1%)	(1, 5.0%)	(4, 6.5%)	
Other	(2, 4.8%)	(0, 0.0%)	(2, 3.2%)	
				
Time since transplant (mean, SD, in years)	(3.6, 4.4)	(2.6, 2.8)	(3.3, 4.0)	0.401
Type of transplant (n, % yes)				0.956
Cadaver	(27, 64.3%)	(13, 65.0%)	(40, 64.5%)	
Living donor	(15, 35.7%)	(7, 35.0%)	(22, 35.5%)	
				
GI complications ^1 ^(n, % yes)				
Diarrhea	(25, 59.5%)	(0, 0.0%)	(25, 40.3%)	
Dyspepsia	(32, 76.2%)	(0, 0.0%)	(32, 51.6%)	
Nausea	(21, 50.0%)	(0, 0.0%)	(21, 33.9%)	
Vomiting	(6, 14.3%)	(0, 0.0%)	(6, 9.7%)	
Abdominal pain/bloating/fullness	(38, 90.5%)	(0, 0.0%)	(38, 61.3%)	
GI bleeding				
Other	(22, 52.4%)	(0, 0.0%)	(22, 35.5%)	
Pyrosis	(6, 14.3%)	(0, 0.0%)	(6, 9.7%)	
Acid reflux	(4, 9.5%)	(0, 0.0%)	(4, 6.5%)	
All others	(12, 28.6%)	(0, 0.0%)	(12, 19.4%)	
				
Severity of GI complaints (n, % yes)				
None	(0, 0.0%)	(20, 100.0%)	(20, 32.3%)	
Mild	(6, 14.3%)	(0, 0.0%)	(6, 9.7%)	
Moderate	(32, 76.2%)	(0, 0.0%)	(32, 51.6%)	
Severe	(4, 9.5%)	(0, 0.0%)	(4, 6.5%)	

p-values were calculated using a two-sided t-test for continuous variables and using a chi-square test for categorical variables

1 Not mutually exclusive

### Internal Consistency Reliability

The estimates of the internal consistency reliability of the GSRS sub-scales were good (range 0.72 – 0.90) with the exception of Abdominal Pain (Cronbach's alpha of 0.63). The GIQLI total and subscale scores demonstrated excellent internal consistency reliability (range 0.78–0.96; not calculated for the Medical Treatment subscale because it is a single item subscale).

### Test-Retest Reliability

Twenty participants were stable from Baseline to Visit 2 (Table 2) and were used to assess the test-retest reliability of the GSRS and GIQLI. ICCs were adequate – above the established cut-off of 0.60 – and statistically significant (p < 0.001) for the GIQLI total score and all subscale scores and also for three of the five subscales of the GSRS (the Reflux subscale had an ICC of 0.57, very close to the cutoff point while the Diarrhea subscale was the exception with an ICC of 0.16).

**Table 2:** Test-retest Reliability (Reproducibility): Score Stability of the GSRS and GIQLI

	**N**	**Mean** ** Test 1**	**Mean** ** Test 2**	**Difference**	**Wilcoxon Rank-Sum** ** test**		**Kendall's Tau** ** correlation**	**ICC**
							
					**Signed** ** rank**	**p-value**		
**GSRS**								
Abdominal Pain	20	1.88	1.73	-0.15	-11.50	0.39	0.64^a^	0.80
Reflux Syndrome	20	1.48	1.13	-0.35	-7.50	0.06	0.67^b^	0.57
Diarrhea	20	1.37	1.63	0.27	12.00	0.25	0.49^b^	0.16
Indigestion Syndrome	20	1.76	1.71	-0.05	-2.00	0.89	0.72^a^	0.93
Constipation	20	1.57	1.50	-0.07	-9.00	0.19	0.73^a^	0.98
**GIQLI**								
Total Score	19	121.64	124.45	2.81	7.00	0.68	0.60^a^	0.87
Symptoms	20	3.47	3.53	0.06	45.50	0.02	0.67^a^	0.78
Emotion	20	3.04	3.31	0.27	11.50	0.57	0.74^a^	0.81
Physical Function	20	3.08	3.15	0.07	6.50	0.64	0.53^b^	0.90
Social Function	20	3.40	3.48	0.08	-0.50	1.00	0.81^a^	0.82
Medical Treatment	19	3.63	3.58	-0.05	28.50	0.23	0.60^a^	0.72

^a ^p < 0.001

^b ^p < 0.05

### Construct Validity

Correlations between the GSRS and the PGWB ranged from r = -0.21 (Diarrhea and Positive Well-being) to r = -0.53 (Indigestion Syndrome and General Health) (Table 3). All GSRS-PGWB correlations were statistically significant (p < 0.0015 or less) except for 8 correlations. Correlations between the GIQLI and the PGWB were higher as both measure similar constructs, ranging from r = 0.43 (Medical Treatment and Positive Well-being) to r = 0.71 (Emotion and Depression) (Table 3). All GIQLI-PGWB correlations were statistically significant (p < 0.001).

**Table 3:** Construct Validity: Correlations* of GSRS and GIQLI Total Scores and Subscale Scores with PGWB Total

	**GSRS Subscales**					**GIQLI Subscales**					
	**Abdominal Pain**	**Reflux Syndrome**	**Diarrhea**	**Indigestion Syndrome**	**Constipation**	**Total Score**	**Symptoms**	**Emotion**	**Physical Function**	**Social Function**	**Medical Treatment**

**PGWB Total Score**	-0.42^1^	-0.32^1^	-0.35^1^	-0.49^1^	-0.44^1^	0.63^1^	0.51^1^	0.68^1^	0.58^1^	0.58^1^	0.47^1^
**PGWB Anxiety**	-0.45^1^	-0.33^1^	-0.33^1^	-0.52^1^	-0.45^1^	0.63^1^	0.51^1^	0.67^1^	0.61^1^	0.54^1^	0.43^1^
**PGWB Depression**	-0.37^1^	-0.22	-0.29	-0.39^1^	-0.37^1^	0.57^1^	0.44^1^	0.71^1^	0.51^1^	0.57^1^	0.49^1^
**PGWB Positive Well-being**	-0.26	-0.21	-0.30	-0.35^1^	-0.30	0.49^1^	0.39^1^	0.57^1^	0.46^1^	0.52^1^	0.38^1^
**PGWB Self-control**	-0.34^1^	-0.24	-0.24	-0.36^1^	-0.40^1^	0.47^1^	0.43^1^	0.53^1^	0.40^1^	0.43^1^	0.36^1^
**PGWB General Health**	-0.45^1^	-0.31^1^	-0.43^1^	-0.53^1^	-0.49^1^	0.61^1^	0.51^1^	0.50^1^	0.60^1^	0.58^1^	0.43^1^
**PGWB Vitality**	-0.39^1^	-0.34^1^	-0.37^1^	-0.45^1^	-0.40^1^	0.59^1^	0.51^1^	0.56^1^	0.57^1^	0.57^1^	0.50^1^

Score and Subscales Scores

* Kendall's tau correlations

^1 ^p < 0.001

### Known Groups Validity

**Figure 1:**
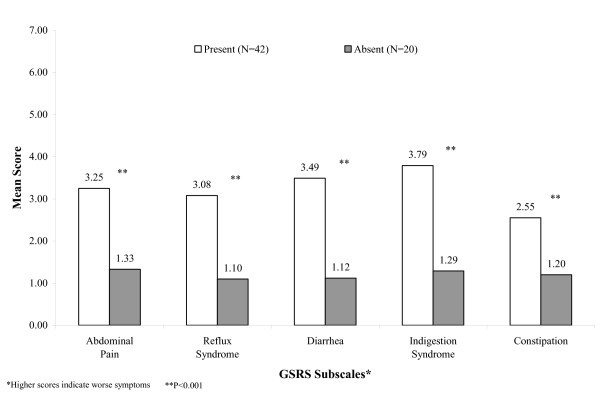
GSRS Subscale Scores by Presence/Absence of GI Complaints.

**Figure 2:**
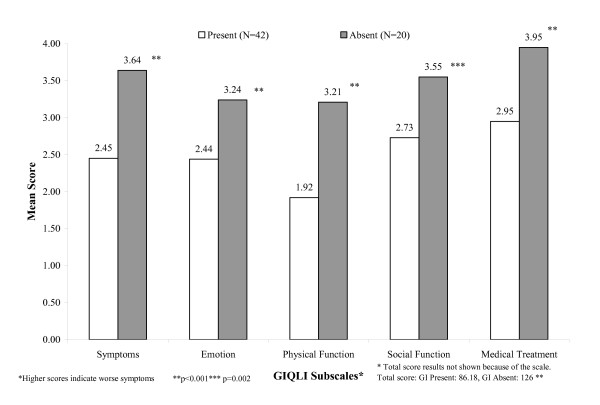
**GIQLI Subscale Scores by Presence/Absence of GI complaints** ***.

Clinical variables used to assess known groups validity were time since transplant; presence or absence of any GI complaint; and severity of GI complaints. Scores on the GSRS and GIQLI were all poorly correlated with the length of time since transplant and no difference by gender was detected (data not shown). All subscales of the GSRS and GIQLI significantly differentiated between patients with and without GI complaints (Figures 1 and 2). The differences were also clinically significant, because in all cases these were above the MID thresholds for each GSRS and GIQLI subscale and total score. Severity level analyses were conducted using four levels: none, mild, moderate, and severe. The GSRS subscales and the GIQLI total score and subscales were able to differentiate among the clinical severity ratings of none, mild, moderate, and severe (overall Kruskall-Wallis test p < 0.001 for each subscale except GIQLI Social Function, Table 4). Many pair comparisons among severity groups were statistically significant (p < 0.0015) and also clinically significant, especially among none and other severity levels but there was no clear consistent gradient between all severity levels.

**Table 4:** GSRS and GIQLI scores by physician's rating of clinical disease severity at baseline

	Groups by rating of clinical severity				Kruskall-Wallis Test of overall differences		
Patient reported outcome instrument and domain	None (n = 20) mean (SD)	Mild (n = 6) mean (SD)	Moderate (n = 32) mean (SD)	Severe (n = 4) mean (SD)	Chi-Square	p-value	Wilcoxon rank-sum tests (pairwise comparisons with p < 0.001)
GSRS Abdominal Pain	1.33 (0.42)	2.61 (0.93)	3.29 (1.23)	3.83 (1.69)	33.75	<0.001	b
GSRS Reflux Syndrome	1.10 (0.26)	2.42 (1.32)	3.09 (1.46)	4.00 (2.12)	29.25	<0.001	b, c
GSRS Diarrhea	1.12 (0.20)	3.61 (1.69)	3.57 (1.56)	2.67 (1.70)	37.21	<0.001	a, b
GSRS Indigestion Syndrome	1.29 (0.33)	3.29 (1.81)	3.76 (1.43)	4.81 (1.68)	38.29	<0.001	a, b
GSRS Constipation	1.20 (0.38)	1.94 (0.65)	2.55 (1.26)	3.42 (2.23)	27.03	<0.001	b
							
GIQLI Total Score	126.00 (10.97)	95.83 (23.22)	87.02 (22.08)	65.00 (37.37)	33.25	<0.001	b
GIQLI Symptoms	3.64 (0.30)	2.65 (0.68)	2.49 (0.57)	1.87 (0.82)	35.31	<0.001	b
GIQLI Emotion	3.24 (0.64)	2.87 (0.70)	2.47 (0.74)	1.60 (0.86)	16.33	<0.001	
GIQLI Physical Function	3.21 (0.61)	2.14 (0.89)	1.92 (0.91)	1.61 (1.62)	22.00	<0.001	b
GIQLI Social Function	3.55 (0.60)	3.04 (0.70)	2.74 (0.96)	2.19 (1.60)	10.22	0.017	
GIQLI Medical Treatment	3.95 (0.22)	4.00 (0.00)	2.94 (1.11)	1.50 (1.91)	21.25	<0.001	b, c

Comparisons not significant unless noted.

a = none vs. mild; b = none vs. moderate; c = none vs. severe;

d = mild vs. moderate; e = mild vs. severe; f = moderate vs. severe.

## Discussion

Psychometric evaluation is an on-going process that incorporates quantitative as well as qualitative testing. No single test result provides information regarding an instrument's psychometric soundness; results of both reliability and validity testing must be weighed together.

The results of this study provide evidence of both the reliability and the validity of the Spanish for Argentina versions of the GSRS and Spanish for Spain GIQLI in patients post renal transplant in South America. These instruments were originally developed for use in the GI area and demonstrated good psychometric characteristics when used with a wide variety of GI diseases and surgical procedures [8,14,16]. In this study, both questionnaires demonstrated extremely good psychometric characteristics, including reliability and validity. Internal consistency reliability of four of the five GSRS subscales was above the 0.70 cut-off for aggregate data. The GIQLI total score and subscales for which a Cronbach's alpha could be calculated were all above 0.70 as well. Four of the five GSRS subscales demonstrated satisfactory (above or very close to the threshold of 0.6) reproducibility over a 4- to 6-week period. The GSRS subscale that did not demonstrate good reproducibility – the Diarrhea subscale – was not the same as the subscale (Abdominal Pain) that fell below the 0.70 cut-off for internal consistency reliability, indicating that there is no pattern of poor reliability. Nevertheless, the low reliability score of the GSRS Diarrhea was surprising. One potential explanation is that the test-retest population may not have been stable in terms of diarrhea at the time of the second measurement. In the PROGIS study, diarrhea was the most frequent GI complaint in both groups after the study started [1], however the occurrence of diarrhea (7.4% in patients with GI events at baseline and 7.1% in patient without GI at baseline) or any GI event (17.7% in patients with GI at baseline and 13.3% in patients without GI events at baseline) was low during the study period. Therefore this new occurrence of diarrhea may not explain the low ICC alone. However, the fluctuations in diarrhea combined with a small sample size could have explained the low ICC. To investigate this hypothesis, a recalculation of this ICC in a larger, stable population was performed by using the stable safety population from the PROGIS study (n = 127) [1]. The ICC for Diarrhea improved to a moderate value of 0.49 (which is nevertheless still below the predetermined cut-off value of 0.6), while the ICC for Abdominal Pain, Indigestion Syndrome and Constipation remained above 0.6 and the ICC for Reflux Syndrome reduced to 0.49. The GIQLI Total Score and subscales all had satisfactory reproducibility. A previous study validated the GSRS and GIQLI in renal transplant patients[2,3]. Due to the cross-sectional study design, test-retest reliability was not evaluated in that research. This work adds to the validity of the GSRS and the GIQLI not only confirming previous findings but by also proving the stability of the scores over time.

Validity of the GSRS and GIQLI was also demonstrated in this study. Construct validity was established through correlations of the questionnaires with another, generic questionnaire, the PGWB. For the GSRS, the highest correlations were seen between the Indigestion Syndrome subscale and the PGWB Anxiety and General Health Subscales. A high correlation was also observed between the GSRS Indigestion Syndrome and the PGWB Total Score and between the GSRS Constipation subscale and the PGWB General Health subscale. The GSRS Diarrhea subscale produced low correlations with the PGWB Depressed Mood and Self-control subscales. The GSRS Reflux Syndrome and Abdominal Pain subscales were also poorly correlated with PGWB Positive Well-being subscale. The patterns of association are also similar those previously reported[14]. The GSRS demonstrates adequate construct validity in this study.

The correlations between the GIQLI and PGWB were higher than those between the GSRS and PGWB, as the GIQLI and PGWB both assess a similar construct (HRQL), whereas the GSRS assesses GI symptoms. The GIQLI Emotion subscale was highly correlated with the PGWB Total score and also with its Anxiety and Depressed Mood subscales. The GIQLI Physical Function and Social Function subscales were highly correlated with the PGWB Total Score and PGWB General Health. The lowest correlations were between the GIQLI Medical Treatment subscale and the PGWB Anxiety, Positive Well-being, and Self-control subscales. Participants received their transplants a little over 3 years prior to enrollment in this study. Medical treatment may not be unusually stressful for them at this point, as they are quite familiar with the healthcare system, their clinicians, and their treatment. Therefore, it is logical that medical treatment would not be associated with anxiety, positive well-being, or self-control.

The GSRS and GIQLI both demonstrated good known groups validity, distinguishing between patients with and without GI complaints. Also, in this South American cohort all the results between the GI and no-GI groups were clinically significant as the differences were above the minimum important clinical difference calculated using the whole PROGIS sample including the South American participants. Both questionnaires were in some instances able to statistically and clinically distinguish among patients with varying GI complication severity demonstrating sensitivity not only to the presence of symptoms but to the severity of those symptoms as well. However, and similar to Kleinman[2,3], clear consistent gradient relationships were not observed.

We acknowledge the relatively small sample size of this study. The significant differences in questionnaire scores observed make those results even more impressive given the small number of participants with mild and severe GI complications. We faced some limitations in terms of the available translations of the instruments for this study. We used the Argentinean Spanish version of the GSRS as the most accurate available GSRS version to represent the study population. However, one center was in Chile, where this version may not have been an optimally culturally adjusted questionnaire. Additionally, we used the Spanish for Spain version of the GIQLI with minimal changes to its wording introduced by the research team based on well known differences in medical terms between Spain and South America. It was the impression that the questionnaires looked acceptable to be used in the study as a high response rate was obtained. However, the use of country-specific versions of the GIQLI and a Chilean-specific version of the GSRS would have been better would these translations have been available.

## Conclusion

The results of the study suggest that the Argentinean Spanish version of the GSRS and the Spanish version of the GIQLI are valid and reliable for use in a post-renal transplant population in South America. These results are a useful addition to the development of patient reported outcomes research in South America. Patients with GI complaints reported poor HRQL and strategies are needed to improve patients' HRQL.

## List of abbreviations

EC-MPS: Enteric-coated mycophenolate sodium; GI: Gastrointestinal; GIQLI: Gastrointestinal Quality of Life Index; GSRS:Gastrointestinal Symptom Rating Scale; HRQL: Health-related quality of life; ICC: Intraclass correlation coefficient; MID: Minimal Important Difference; MMF: mycophenolate mofetil; PGWB: Psychological General Well-Being; PROGIS: Patient Reported Outcomes in Renal Transplant Patients with and without Gastrointestinal Symptoms.

## Competing interests

Gerardo Machnicki is an employee of Novartis Pharmaceuticals Corporation and Alcira Otero is an employee of Novartis Argentina SA.

## Authors' contributions

JP, LG, AML, CR and RS were involved in carrying out the study and reviewed the manuscript. GM created the project, reviewed the study design, supervised the data analysis and wrote the manuscript. AO created the project, reviewed the study design and data collection and reviewed the manuscript. MKM supervised the study design, data collection and statistical analyses and wrote the manuscript.
